# Arboviruses and Muscle Disorders: From Disease to Cell Biology

**DOI:** 10.3390/v12060616

**Published:** 2020-06-05

**Authors:** Claudia Filippone, Vincent Legros, Patricia Jeannin, Valérie Choumet, Gillian Butler-Browne, Jim Zoladek, Vincent Mouly, Antoine Gessain, Pierre-Emmanuel Ceccaldi

**Affiliations:** 1 Unit of Epidemiology and Physiopathology of Oncogenic Viruses, Institut Pasteur, 75015 Paris, France; vincent.legros@ens-lyon.fr (V.L.); patricia.jeannin@pasteur.fr (P.J.); jim.zoladek@pasteur.fr (J.Z.); antoine.gessain@pasteur.fr (A.G.); 2 Centre National de la Recherche Scientifique (CNRS) UMR 3569, 75015 Paris, France; 3 Virology Unit, Institut Pasteur de Madagascar, Antananarivo 101, Madagascar; 4 Université de Paris, 75013 Paris, France; 5 CIBU Unit, Institut Pasteur, 75015 Paris, France; valerie.choumet@pasteur.fr; 6 Sorbonne Université, Inserm, Institut de Myologie, UMRS 974, Center for Research in Myology, 75013 Paris, France; gillian.butler-browne@upmc.fr (G.B.-B.); vincent.mouly@upmc.fr (V.M.)

**Keywords:** arbovirus, myopathy, myositis, pathophysiology, myoblasts, alphavirus, flavivirus

## Abstract

Infections due to arboviruses (arthropod-borne viruses) have dramatically increased worldwide during the last few years. In humans, symptoms associated with acute infection of most arboviruses are often described as “dengue-like syndrome”, including fever, rash, conjunctivitis, arthralgia, and muscular symptoms such as myalgia, myositis, or rhabdomyolysis. In some cases, muscular symptoms may persist over months, especially following flavivirus and alphavirus infections. However, in humans the cellular targets of infection in muscle have been rarely identified. Animal models provide insights to elucidate pathological mechanisms through studying viral tropism, viral-induced inflammation, or potential viral persistence in the muscle compartment. The tropism of arboviruses for muscle cells as well as the viral-induced cytopathic effect and cellular alterations can be confirmed in vitro using cellular models. This review describes the link between muscle alterations and arbovirus infection, and the underlying mechanisms.

## 1. Introduction

The arboviruses (arthropod-borne viruses) represent one of the best examples of viral emergence [1], since their burden has dramatically increased over the last few years [2,3,4,5]. Their emergence and re-emergence at the global level may be caused by several factors: environment (climate change, deforestation); virus evolution, such as acquisition or increase of virulence (genetic changes); and vector properties (geographical expansion of mosquito vectors, increase in mosquito anthropophily), human host (human behavior, globalization, overpopulation.) [6].

Arboviruses include taxonomically different viruses, having in common the mode of transmission occurring through an arthropod vector such as mosquito or tick. Arboviruses include up to eight viral families, with around 500 virus species described, 50 of which are considered pathogenic for humans. Arboviruses are traditionally classified by the symptoms which are linked to the infection in humans, such as hemorrhagic, encephalitic, or arthrogenic [6].

Symptoms associated with acute infection of most arboviruses are often described as “dengue-like syndrome”, including fever, rash, conjunctivitis, arthralgia, and muscular symptoms such as myalgia, myositis, and rhabdomyolysis. Myalgia, defined as muscle pain, is a non-specific symptom that can be found during the dengue-like syndrome of acute infection of almost all arboviruses. Myopathy is generally defined as a pathological alteration of the muscle; many different types of myopathies exist, with a variable level of clinical outcome, from benign to lethal. Rhabdomyolysis can have many causes, including drug or other toxicity, and can be associated to renal failure. Inflammatory myopathies are defined as myositis. Several types of myositis have been defined, according to the mechanisms leading to their development [7]: idiopathic inflammatory myopathy, inclusion body myositis, dermato-myositis/polymyositis, necrotizing myositis, other myositis of auto-immune origin, or neurogenic myositis. In viral myositis, inflammation is associated with the migration of inflammatory cells following viral infection, commonly defined as the immune cell infiltrate. For myositis related to autoimmune disease, it is not yet clear how viral infection interplays with the development of an auto-immune response [8].

Several infectious agents, parasites, bacteria, and viruses can provoke myalgia, acute myositis, chronic myositis, or rhabdomyolysis [9,10,11]. Among them, viral infections mainly associated with muscle failure are: influenza virus, HIV, HTLV-1, hepatitis viruses (B and C), coronavirus, enteroviruses, adenoviruses, cytomegalovirus, Epstein–Barr virus, Torque teno virus, parvovirus B19, and arboviruses, especially, among these latter, those belonging to *Togaviridae* and *Flaviviridae* families [8].

The scope of the proposed review is to provide a description of those arboviruses which have a tropism for muscle cells, and which are associated with pathological alteration of the skeletal muscle. We will especially focus on the consequences of infection in humans (ex vivo), and on both in vivo and in vitro studies aimed to decipher the pathophysiology of the viral infection in this organ.

Existing literature was first searched in PubMed database by using keywords ‘arbovirus’ alternatively combined with ‘muscle’, ‘myositis’, ‘myocarditis’, ‘rhabdomyolysis’, and ‘human’, ‘animal model’, ‘mice’, ‘model’, and ‘cell’, respectively. Then, the same was done with distinct genus’ and species’ names of arbovirus (e.g., *alphavirus*, *flavivirus*, Chikungunya, Zika, dengue). Selection of published works was made according to more detailed provided descriptions: for human studies, included works were chosen according to the suggestion of causality factor (etiology) between arbovirus infection and muscle failure in clinical studies; for in vivo and in vitro studies, the strategy for selecting publications was done according to the indication of occurrence of arbovirus infection in muscle compartment. In very few cases, responding to such requirements, works found on Google Search were also included.

## 2. Muscular Symptoms Associated with Arboviral Infection

Among those arboviruses which are a threat to human health, alphaviruses and flaviviruses are the most frequent causes of recent worldwide epidemics.

In humans, arboviruses are associated with several diseases; according to a traditional classification, they can be described as hemorrhagic (e.g., dengue virus (DENV)), encephalitic/neurotropic (e.g., West Nile virus (WNV), Zika virus (ZIKV)) or arthrogenic (e.g., Ross River virus (RRV), Chikungunya virus (CHIKV), Sindbis virus (SINV)). However, overlapping symptoms are often observed, thus making it difficult to discriminate these diseases into specific groups.

A common feature to several arboviruses is the virus-induced alteration of the musculo-skeletal system, with a clinical relevance of both arthropathies and myopathies. While arthritis and arthropathies associated with arboviruses have been largely studied [12], overall for alphaviruses RRV, SINV, and CHIKV [13,14,15,16,17,18,19,20,21,22], little attention has been given in the past to muscle as an organ potentially targeted by arboviruses. However, as reviewed by Suhrbier et al. [12], the frequency of myalgias during infection by arthrogenic viruses, such as CHIKV, RRV, Barmah Forest virus, SINV, O’Nyong-nyong virus and Mayaro virus, range between 40% and 90% among the patients. In a study conducted in Australia, RRV infection in humans was found to be associated with muscle pain in 66.7% of infected patients (*N* = 255) [23]. Similarly, as reviewed in Dupuis-Maguiraga in different studies performed during CHIKV outbreaks in La Réunion and Indian Ocean area, myalgia was reported in up to 84% of CHIKV-infected patients [24]. In a comparative study concerning CHIKV and dengue virus infection in Gabon, myalgia was reported among the clinical symptoms in more than 70% of infected patients, 195/270 for CHIKV, and 40/53 for DENV, respectively [25]. A recent study conducted in French Guiana, points out the occurrence of muscle pain in 66.1% among CHIKV-infected patients (111, *N* = 168), and 80.9% among dengue-infected patients (366, *N* = 452) [26]. Concerning ZIKV, myalgia has been reported in 44% of infected patients (131, *N* = 297) in French Polynesia, and 60% (121, *N* = 203) in Martinique [27,28].

Muscle pain does not only concern the acute phase of the infection, as it can persist beyond it [29]; in a follow-up study on DENV infected patients, more than 60% (20, *N* = 31) of patients still suffered from myalgia, two months following their hospitalization [30]. In addition, some arboviral infections may lead to long-term arthralgias and myalgias as shown for example with Sindbis virus, where sequelae such as myalgia are still observed six months after infection [31], and arthralgias were still present in 25% of infected patients after three years [32].

Of note, myositis may occur following infection from arthrogenic (e.g., RRV, CHIKV, SINV), encephalitic/neurotropic (e.g., WNV, ZIKV, tick-borne encephalitis virus (TBEV)), or hemorrhagic arboviruses (e.g., DENV). Neurogenic myositis was reported to be associated with WNV, CHIKV, and ZIKV, as a consequence of polio-like, transverse myelitis, and Guillain-Barré syndromes, respectively [33,34,35]. Myositis has been described in one case report during CHIKV infection [36], and in two CHIKV positive patients with detection of infected cells (satellite cells, i.e., muscle progenitor cells) in muscle, in one of them several months post-infection [37]. Severe myalgia and acute myositis have also been described in DENV-infected patients. For example, in one study, 15 cases were reported [38], in another seven cases, two of them with a lethal outcome [39]. In a case report, myositis persisted for two months in a DENV-infected patient [40]. Arboviral-induced myositis has also been reported during childhood after DENV [38,39] or TBEV [41] infection. A more severe outcome occurring during some arboviral infections is rhabdomyolysis, as reported in DENV, WNV, CHIKV, and tick-borne Alkhurma hemorrhagic fever virus-infected patients [42,43,44,45,46]. Interestingly, in a large-scale study during the 2014–2015 CHIKV epidemics in French Guiana, three of the 285 infected hospitalized patients exhibited rhabdomyolysis [47]. Of note, such arboviral induced rhabdomyolysis can lead to acute renal failure with possible fatal outcome [45,48].

Another type of myositis that does not concern skeletal muscle is myocarditis (i.e., inflammation of the myocardium). Indeed, arboviruses such as CHIKV, ZIKV, and DENV can provoke myocarditis in some cases [49,50,51,52]. In a large scale pediatric study in Colombia, among 102 DENV infected patients, 11 of them (10.7%) presented myocarditis with one fatal case [53], and in a study conducted in China, the prevalence of myocarditis in hospitalized confirmed dengue patients reached 11.28% (201 out of 1782 patients) [54].

## 3. Muscle Alterations and Arboviral Myotropism in Humans

Detection of arbovirus genomes or antigens in muscle biopsies supports the link between virus infection and muscle phenotype. However, in some cases, the inflammatory state or alteration in muscle is reported while infection is confirmed only at the systemic level.

Only very few studies are available ex vivo, since arboviral myositis are often transient, thus limiting the availability of muscle biopsies (Table 1).

Concerning alphaviruses, in the study from Sane and colleagues, although no SINV antigens could be detected on the muscle biopsy from an infected patient by immunohistochemistry, ongoing muscle regeneration was demonstrated by an increased number of internal nuclei, and an increased immunoreactivity for neonatal Myosin Heavy Chain [31].

In muscle biopsies obtained from two confirmed CHIKV-infected patients with myositis, during the epidemic outbreak in the French island of La Réunion in 2006, infiltrating cells were mostly identified as monocytes/macrophages (CD68 staining) and T-cells (CD3 staining), and vacuolization of muscle fibers and fibrosis were detected. Although viral antigens are rarely searched for, CHIKV antigens were detected in this study in satellite cells (i.e., myoblasts), but not in muscle fibers [37].

Concerning flaviviruses, anatomopathological studies in DENV-infected patients with arboviral-induced myositis indicate perivascular mononuclear infiltrates [38] and fibrosis [39]. Of note, concerning DENV-induced myocarditis, inflammatory markers such as monocyte chemoattractant protein-1 (MCP-1) could be found in myocardial endothelial cells, cardiac interstitial cells, and myocardial myoblasts, and DENV antigens could be detected in cardiomyocytes, myocardial interstitial cells, and endothelial cells [59].

Other studies have also reported muscle alteration in human specimens [55,56,57,58,60,61], although the presence of the virus is not always demonstrated.

Table 1 illustrates cases of muscle ex vivo studies from infected human patients.

## 4. Animal Models of Arboviral Myopathies

In the context of muscular skeletal system alterations provoked by arboviral infection, several animal models have been developed (Table 2), suggesting that muscle is a target of viral infection [62,63,64,65].

### 4.1. Ross River Virus

Ross River virus is considered as the prototype among the arthrogenic alphaviruses, and is one of the most studied arboviruses for muscular alterations. Several works have provided models to study the pathogenesis of RRV (Table 2). Murine models have been used to investigate both viral infection of the muscular tissue and related inflammation causing tissue damage and development of myositis [66,67,68,69]. RRV has been shown to provoke myositis in infected outbred mice [66], and inflammation was detected in skeletal muscle as well as in bones and joints [67]. In a mouse model, lacking a functional T-cell response, described by Burrack and colleagues, histological analysis confirmed inflammation and, most interestingly, viral RNA was detected in muscle tissue with an important titer, which confirms the role of T-cells in the control of RRV infection in a tissue specific manner [70].

In an RRV mouse model of arthritis (C57BL/6 wild type mice), inflammation and tissue damage were studied in muscle in relation to the role of macrophages in establishing disease [71]. Recently in a murine model, live monitoring has been performed during acute and long-term RRV infection by in vivo imaging using a bioluminescent virus [72]. Interestingly, in this model, active RRV replication could be detected in the gastrocnemius and masseter muscles, as well as in the heart [72].

### 4.2. Chikungunya Virus

Pathogenesis of the inflammatory myopathy (myositis) related to CHIKV infection was studied in mice and non-human primates (NHPs) [63,73]. Several mouse models, such as IFNAR−/−, new-born CD-1, C57BL/6, and others, were shown to develop myositis upon infection with CHIKV [63,73,74,75]. Inoculation of CHIKV in mice provoked myositis, together with arthritis and tenosynovitis [75]; tissue damage and inflammation in both muscle and joint were observed in parallel with viremia [65]. In the model developed by Dhanwany and colleagues, based on new-born mice, muscle degeneration related to viral infection was reported [74]. Infected mice could develop a muscle pathology, with cell infiltrates and viral replication within the muscle, although specific target cells were not characterized [74]. In the model described by Nair and colleagues which was focused on the role of interferon in controlling viral replication, the presence of viral RNA was observed in muscle cells by in situ hybridization [76]. In addition, a co-staining of CHIKV envelope protein E2 antigen and fibroblast vimentin was observed [76].

**Table 2:** In vivo muscle tropism of arboviruses. Arboviruses associated with muscle impairment in animal models following infection with description of tropism (in vivo).

Virus (Family, Genus)	Virus (Species)	Animal Study (*N*)	Alteration, Histopathology/Viral Markers, In Vivo Tropism (Muscle)	References [*n*°]
***Togaviridae Alphavirus***	**Ross River virus**	mice C57BL/6N (3)	viral replication *	[72]
		mice C57BL/6J mice B6-SJL mice Irf1^−/−^ C57BL/6 (2–3)	viral antigens, infectious virus *	[76]
		outbred mice mice macrophage^(−/−)^ (3–4)	disruption, inflammatory infiltrate, pro-inflammatory cytokines/viral replication *	[66]
		outbred mice CD-1 adult mice C57BL/6J mice RAG-1^(−/−)^(3–6)	inflammatory infiltrate/viral RNA, viral replication *	[67]
		mice C57BL/6J mice C3^−/−^ mice RAG-1^(−/−)^ (3–6)	damage, inflammatory infiltrate/viral RNA, viral replication *	[69]
		mice C57BL/6J mice MIF^−/−^ (5)	disruption, inflammatory infiltrate/viral replication *	[71]
		mice C57BL/6 mice MBL-DKO mice C1q^−/−^ mice fB^−/−^ (2–3)	disruption, inflammation/viral RNA, viral replication *	[77]
		mice C57BL/6J mice CD74^−/−^ mice MIF^−/−^ (2–6)	disruption, inflammatory infiltrate/viral replication *	[78]
		adult mice C57BL/6J mice TLR7^−/−^ mice Myd88^−/−^ (5–8)	disruption, inflammatory infiltrate/infectious virus *	[68]
		mice C57BL/6J mice CD8α^−/−^ mice Rag1^−/−^ (2–6)	inflammatory infiltrate/viral replication *	[70]
		mice C57BL/6J mice CCR2-DTR mice C57BL/6J Irf3^−/−^, Irf7^−/−^ (3)	inflammation/viral replication *	[79]
	**Chikungunya virus**	new-born mice (3–8)	structural degeneration, necrosis, inflammatory infiltrate, inflammatory cytokines/viral antigens, viral replication *	[74]
		young mice C57BL/6J (3–4)	necrosis, inflammatory infiltrate/viral replication *	[75]
		new-born mice CD-1 young mice ICR (3–4)	degeneration, necrosis, fibrosis, dystrophic calcification, inflammation/viral antigens, viral replication *	[65]
		mice C57BL/6J mice B6-SJL mice C57BL/6J Irf1^−/−^ (2–3)	inflammation/viral antigens, viral RNA, infectious virus *	[76]
		outbred mice OF1 new-born mice C57BL/6 mice 129s/v mice IFN-α/ßR^−/−^ 129s/v (4)	myofiber necrosis, inflammatory infiltrate/viral antigens #, viral replication *	[64]
		mice C57BL/6 mice IFNAR^−/−^ (4–5)	disruption, necrosis, inflammatory infiltrate/viral burden ° *	[80]
		mice C57BL/6J mice CD74^−/−^ (2–6)	inflammation	[78]
		Hamster (5–14)	necrosis, inflammation (myositis, tenosynovitis, myocarditis, fasciitis)/virus burden *	[81]
		Zebrafish (3–5)	viral antigens	[82]
		cynomolgus macaque (12)	focal necrosis, inflammatory infiltrate/viral antigen-RNA (persistent at 1.5 months post-inoculation ^§^), viral replication *	[83]
		rhesus macaque (2–6)	fiber necrosis, mononuclear infiltrate (perimysium)/viral RNA (persistent at 21 dpi)	[84]
	**Sindbis virus**	neonatal mice CD-1 (3)	necrosis, inflammatory infiltrate/viral RNA	[85]
	**Barmah Forest virus**	Swiss outbred mice mice C57BL/6 (3–5)	inflammatory infiltrate/viral replication *	[86]
	**Mayaro virus**	mice Balb/c (3)	fiber degeneration, inflammation, MCP-1/viral replication *	[87]
		mice SV129 mice IFNAR^−/−^ mice C57BL/6 mice RAG^−/−^ (4–6)	damage, necrosis, inflammation/infectious virus *	[88]
***FlaviviridaeFlavivirus***	**Zika virus**	mice 129Sv mice AG129 (2–4)	multifocal myofiber degeneration, necrosis, nuclear rowing, attempted regeneration, infiltrate/viral replication *	[89]
		rhesus macaque (7)	viral RNA (persistent at 35 dpi)	[90]

* Viral replication occurring in muscle, measured as viral load (qRT-PCR) or titration of infectious virus (focus-forming unit (ffu) or plaque forming unit (pfu)). # Fibroblasts of epimysium and perimysium, and few satellite cells of skeletal muscle. ° Myofibers. ^§^ Muscle macrophages.

In the immunodeficient IFNAR−/− mice, successfully used as a model for CHIKV infection [64], CHIKV was shown to replicate in the epimysium (i.e., muscle fascia) of skeletal muscle. Fibroblasts, myofibroblasts, and, less frequently, satellite cells but not myotubes were shown to be infected [64]. Infection of myofibers were detected in a single study, in a neonatal murine model but only for one viral strain [80]. Recently, a mouse model was also employed to elucidate mechanisms of pathogenesis of CHIKV in muscle by using an engineered virus, which allows one to follow viral replication in muscle cells [91].

Concerning CHIKV, other animal models have shown muscle involvement during infection. In a hamster model, development of myositis has been reported [81]; in a zebrafish model, developed for the study of the immune response, muscle was confirmed to be infected by CHIKV, although identification of the specific target cell type could not be clarified [82]. In addition, in a non-human primate (NHP) model, such as a macaque infected with CHIKV, viral RNA was detected in several tissues including muscle [83,84].

### 4.3. Other Alphaviruses

Other reports concern different alphaviruses. Interestingly, in a new-born mouse model of SINV infection, viral RNA was also detected in muscle [85]. In the case of Barmah Forest virus, this latter has been reported to cause myositis and replicate in muscle, even if to a lower extent than RRV [86]. More recently, a mouse model of myositis (and arthritis) induced by Mayaro virus (MAYV) has been developed, showing virus-induced muscle inflammation, fiber degeneration, and regeneration similar to what has been observed for CHIKV and RRV. Moreover, viral replication was shown to occur within muscle tissue [87]. IFNAR−/− and RAG−/− mouse models were employed to investigate the role of both type I interferon response and adaptive immunity in MAYV replication and muscular inflammation [88]. Finally, another (non-human) alphavirus, not transmitted by arthropods, i.e., the sleeping disease virus that infects salmonids, has been shown to target selectively the satellite cells in muscle of rainbow trout (*Oncorhyncus mykiss*) which had been experimentally infected [92].

### 4.4. Zika Virus

Since the re-emergence and associated epidemics of ZIKV in 2016, many studies have been carried out to develop animal models [62,93], mainly based on mice and NHPs. Indeed, these models have been crucial to clarify the large tropism of ZIKV, not only towards the central nervous system, but also for other tissues, including muscle. Mouse models of ZIKV infection include adult and neonate animals, either immunocompetent or immunocompromised, as recently reviewed by Morrison and Diamond [62]. The model based on AG129 mice deficient in alpha-beta interferons and in IFN-gamma receptor, showed degeneration of muscle fibers with infiltration of inflammatory cells upon infection, and presence of viral RNAs in the muscle tissues [89].

Concerning NHPs, the macaque seems to be an appropriate model, especially to detect the persistence of ZIKV RNA in muscle [90]. Indeed, in this study, after ZIKV infection of a macaque model, in addition to the observation of viremia, the viral genome could be detected by RT-PCR for up to 35 days post-infection in different tissues including muscle and joint, although it is not clear if replication occurred in muscle cells or in cells involved in inflammation [90].

## 5. In Vitro Models of Muscle Cell Infection

Cell culture still remains an essential tool of investigation in virology. It is well-known that many tissues can harbor arboviruses, but there is still a need to more precisely clarify the tropism for the cells of the musculo-skeletal system, by defining which specific cells are the targets of the viruses, and whether the tropism is restricted by cell or viral factors. Table 3 illustrates the cell models which have been developed.

### 5.1. Chikungunya Virus

**Figure 1:**
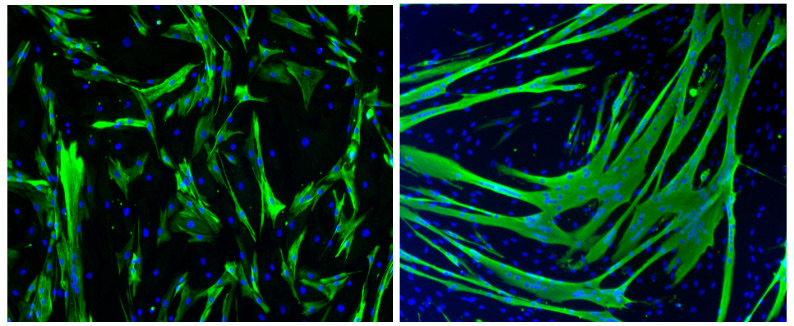
Study model of human primary muscle cells. Human primary mononucleated myoblasts (left panel), cultured in vitro, are differentiated into multinucleated myotubes (right panel). Muscle cells are labeled by an anti-desmin mouse antibody, and a secondary goat anti-mouse monoclonal antibody (green staining), and nuclei by DAPI staining. Magnification: 200×.

Since the emergence of the CHIKV, its tropism has been investigated in several cell types [101], although initially not a lot of attention was given to muscle as a possible target. One of the first studies, carried out by our group to elucidate the tropism of CHIKV for muscle cells, showed the presence of viral antigens ex vivo on biopsies from two infected patients [37] (see above). In vitro, human primary myoblasts have been shown to provide a very useful tool for the study of viral infection, since they can be differentiated into myotubes (Figure 1), thus allowing one to assess the susceptibility to viral infection according to cell state, undifferentiated muscle cells compared to differentiated myotubes.

**Figure 2:**
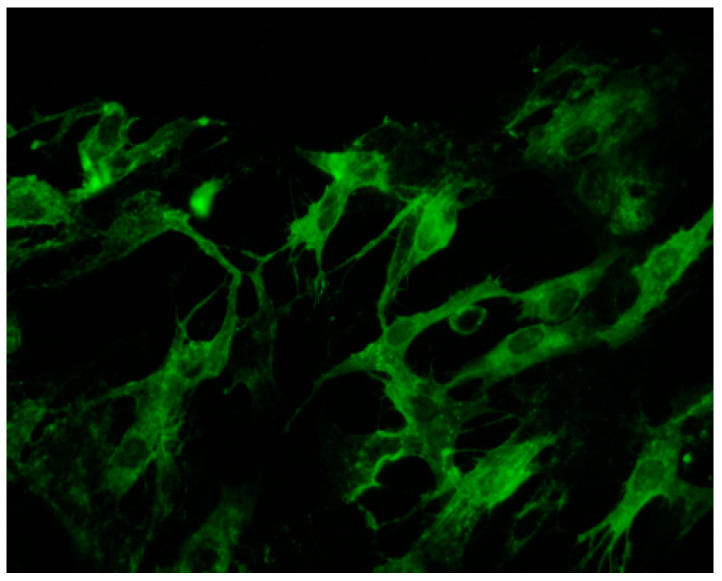
In vitro infection of human myoblasts by Chikungunya virus (CHIKV). Human myoblasts in culture were inoculated with CHIKV (strain from La Reunion outbreak, 2006), at a multiplicity of infection of 1, as described in Ozden et al. (2007). Twenty-four hours later, infection was assessed by immunofluorescence using a mouse serum directed towards CHIKV, and a secondary goat anti-mouse monoclonal antibody coupled to FITC. Magnification: 400×.

In our previous study, productive infection has been observed on myoblasts (Figure 2), without any infection of differentiated myotubes, thus suggesting that cell differentiation could be critical for susceptibility and permissiveness of viral infection [37].

Indeed, muscle satellite cells together with fibroblasts, osteoblasts, endothelial cells, and macrophages would be among the cells targeted during acute infection of CHIKV [102,103]. Hussain et al. confirmed this model for pathogenesis based on human primary myoblasts [94]. It has also been suggested that some isolates used during infection may be more virulent for myoblasts than others [95], or can play a role in the gain of susceptibility of myofibers [80]. However, these observations are in contrast with the previous observations from Ozden and colleagues on muscle cells, as well as the study from Sourisseau et al. that showed the infection patterns were not affected by the type of CHIKV strain on several cell lines [37,101].

Interestingly, the study from Ozden and colleagues, based on primary human muscle cells cultures, provided an interesting model that could be further used to investigate the factors involved in the restriction to CHIKV infection or to search for viral inhibitors [104]. Of note, a similar study carried out on another alphavirus, the sleeping disease virus of salmonids, although not related to arboviruses, showed that primary cultures of trout myoblasts were productively infected [92], thus inviting one to speculate on intrinsic general features of alphaviruses favoring a tropism toward the muscle compartment, independently on the infected species.

A muscle cell line derived from a rhabdomyosarcoma SJCRH30, although less physiological, has been used to confirm that CHIKV is able to infect and replicate in muscle cells [96]. The same cell line was recently employed to assess CHIKV entry in muscle cells via macropinocytosis [105]. In another study, human muscle cell monolayer (RD cell line) showed to support CHIKV replication [97].

### 5.2. Sindbis Virus

Sindbis virus, one of the most common and studied arbovirus in Europe [29], has been shown to successfully infect both primary human myoblasts and myotubes in vitro, with detection of viral antigens and observation of cytopathic effects on infected cells [31]. However, it has not yet been fully elucidated as to whether different strains of SINV are equally pathogenic in muscle [29,31].

### 5.3. Zika Virus

In a study from Chan and colleagues, a large panel of cell lines were tested for infection by and culture of ZIKV. Among them, a rhabdomyosarcoma cell line (RD) was shown to be productively infected by ZIKV [98].

A recent report from our group indicates high susceptibility of human primary myoblasts to ZIKV infection, whereas myotubes seem to be resistant (Legros V et al., accepted for publication, PLOS NTDs).

### 5.4. Dengue Virus

Human muscle satellite cells have been shown to be susceptible to DENV, independently from the serotype employed [99], as shown by detection of both viral RNA and antigens following infection. Additional studies have more recently demonstrated that DENV is able to productively infect differentiated myotubes [59]. Permissiveness of a rhabdomyosarcoma cell line (RD) to DENV-2 has also been demonstrated [98].

In addition to these studies, another flavivirus, WNV, has been suggested to be able to infect human embryonic myoblasts in vitro [100].

## 6. Possible Mechanisms of Pathophysiology of Arbovirus Associated Muscle Alterations

Pathophysiology and pathogenesis of arbovirus-associated myopathies, such as myositis, should then be considered as a consequence of host–virus interactions following viral infection.

Cell permissiveness to a virus in a cell compartment is defined by the presence of specific receptors and entry pathways, but also by the presence of cellular proteins favoring or restricting viral replication, and of other cellular determinants. The rationale of the studies that have been carried out in skeletal muscle over the last few years have aimed at exploring the mechanisms behind the frequent symptomatology of pain, inflammation, and degeneration of muscle following arbovirus infection.

Animal models are of great importance to establish the occurrence of interactions between arboviruses and muscle cells. Mouse models helped identify the participation of inflammatory cells such as macrophages in developing arthritis and myositis and to establish the damage, and the role of pro-inflammatory factors such as tumor necrosis factor (TNF)-α, MCP-1, and interferon (IFN)-γ.

**Figure 3:**
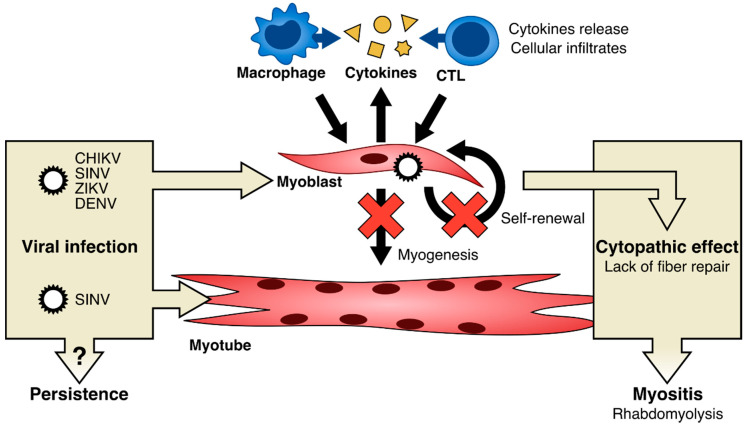
Proposed model for arboviral-induced muscle cell alteration leading to muscle disorders. Muscle cell alteration may include infection of myoblasts and/or myotubes, according to different Arboviruses. Infection may induce the release of cytokines (e.g., TNF-α, MCP-1, IFN-γ, MIF, IL-6, Rantes…), macrophage recruitment and CD8+ T lymphocytes activation. These events may lead to cytopathic effect, dysregulation of myogenesis, and may inhibit the self-renewal of muscle progenitor cells. Alternatively, or in addition, cytotoxicity may send molecular signals to attract inflammatory cells. Abbreviations. CHIKV: Chikungunya virus; SINV: Sindbis virus; ZIKV: Zika virus; DENV: dengue virus; CTL: cytotoxic T lymphocyte.

Indeed, mouse models have played a key role in deciphering disease pathogenesis, suggesting that, in most cases, together with virus-induced cell damage, the myopathy is caused by the inflammatory cell infiltrate (Figure 3), as it is the case for development of arthritis.

One study from Lidbury et al., carried out in a murine model infected with alphaviruses (RRV), pointed out a secretory function of macrophages in establishing myositis [66], with a mechanism comparable to the one involved in arthritis. Thus, macrophages play a critical role in the development of the disease (Figure 3).

Another study suggested the role of macrophages for development of myositis associated with RRV [71] by the action of macrophage migration inhibitory factor (MIF), a factor indicating the activation of macrophages, as shown in a mouse model in which a higher level of MIF was correlated with higher inflammation (by modulation of pro-inflammatory cytokines) and severity of disease (more cell infiltrates and more muscle disruption). In a second study by the same author, the role of CD74 was also demonstrated, in particular as regulator of apoptosis of recruited cells at the site of inflammation [78]. Inflammation mediated by monocyte has also been investigated by studying CCR2 depleted mice [79]. For RRV, it was also suggested that the pathogenesis of myositis may be related to complement activation pathways [69,77]; in particular, the mannose binding lectin (MBL) would affect the expression of inflammatory mediators within the RRV infected muscle, thus contributing to tissue destruction and disease [77]. In the study from Nair and colleagues, the use of a mutant mice (Irf1−/−) depleted for interferon regulatory factor 1 (IRF-1) and infected with RRV and CHIKV, confirmed the role of the interferon early response to control virus replication and its role in virus spreading, inflammation and finally the pathogenesis [76]. When the IFN response was blunted, the muscle pathology was more severe. This study suggested an interplay between viral infection and inflammation and the role of the IFN response for establishing pathogenesis in muscle [76]. Similar behavior was observed for type I interferon response during MAYV infection [88]; in the same study, the role of adaptive immunity was investigated, suggesting a specificity for such alphavirus among the others [88].

RRV infection in muscle tissue of infected mice appears to be controlled by CD8+ cytotoxic cells (Figure 3). Moreover, virus-specific cytotoxic CD8+ seemed to be active in muscle rather than in joint [70]. A recent study employing an engineered virus demonstrated the role of IL-6 in inflammation and pathogenesis of CHIKV in muscle [91].

Proteomic studies of arbovirus infection have also been developed to study pathogenesis in muscle: a study using a mouse infected with CHIKV showed that muscle damage is correlated with a variation of the cellular proteome following infection, especially concerning proteins related to energy and iron metabolism, stress (heat-shock proteins), structure (actin, vimentin, myosin) and inflammation [74]. In particular, high levels of pro-inflammatory mediators such as IL-6, MCP-1, MCP-3, Rantes, and TNF-α were observed [74]. Proteomic studies have been performed on a rhabdomyosarcoma muscle cell line (SJCRH30) infected with CHIKV; in this work Issac and colleagues proposed that some cell proteins, such as vimentin (a cytoskeleton protein involved in maintaining cell structure), could play a role in regulating CHIKV pathogenesis, by interacting with the viral protein nsP3, a part of the CHIKV replication complex [96]. Vimentin would support CHIKV replication, as confirmed by inhibition of infection following silencing of its expression (siRNA). Interestingly, vimentin is expressed in myoblasts, but its expression is down-regulated in myotubes. However, the limit of this study stays in the use of a cell line rather than primary cells, with the latter being more appropriate to conclude on the validity of the proposed mechanism in muscle compartment in vivo.

Among the cellular models, primary human myoblasts represent an appropriate model for the study of mechanisms involved in the pathogenesis of myositis. Myoblasts infected with CHIKV [94] were compared by transcriptomic analyses with non-infected cells; this study suggests the critical function of genes related with apoptosis, innate immunity, and inflammation, that could play a role during viral replication and thus affecting the clinical outcome [94].

Concerning DENV, Warke and colleagues performed a gene expression study on infected myoblasts, and identified genes potentially involved in the immune control of infection [99]. They observed an upregulation of genes involved in IFN antiviral response and others (e.g., IFN response factor 7 (IRF-7), melanoma-derived antigen 5 (MDA-5, …)), although the exact mechanisms of the immunopathogenesis are still not clear [99].

In another study, DENV productively infected heart as well as skeletal muscle cells, and an increase in pro-inflammatory cytokine IP-10 was observed in infected myotubes [59].

Another mechanism potentially involved in arbovirus-induced myositis is the direct infection of muscle cells (Figure 3). Up until now, only one report has clearly demonstrated the infection of muscle by arboviruses in human skeletal muscle biopsies; during the chikungunya outbreak in La Réunion, in 2006, immunohistochemistry studies on muscle biopsies from two infected patients revealed that satellite cells were selectively infected by CHIKV, while myotubes were not [37]. However, as stated before, in animal models, viral RNAs/antigens could be retrieved in the muscle compartment, as shown for RRV [67,69,72,76,77,78], CHIKV [64,65,74,76,80,82,83,84], SINV [85], ZIKV [89,90], and viral replication was observed in muscle for RRV [66,67,68,69,70,71,72,76,77,78,79], CHIKV [64,65,74,75,76,80,81,83], Barmah Forest virus [86], MAYV [87,88], ZIKV [89]. Interestingly, in CHIKV animal models, satellite cells were found to be infected [64], as well as myofibroblasts [64], and, to a lesser extent and only for one viral strain, myofibers [80]. However, whether infected satellite cells can transfer the viral genome to fibers in vivo during myonuclear turnover or regeneration is not clear. Of note, when human primary myoblasts are infected in vitro by arboviruses, a clear cytopathic effect can be observed; in dengue-infected myoblasts, a pronounced cytoplasmic vacuolization has been reported [99], as well as in other cell types during flavivirus infection [106]. A strong cytopathic effect was also detected in myoblasts infected by CHIKV [37,94,95], as well as by other alphaviruses: in the case of SINV, a disorganization of the actin cytoskeleton as well as cell rounding and detachment was observed [31]; concerning the rainbow trout alphavirus, a cytopathic effect was also observed, characterized by scattered foci of rounded cells [92]. Since satellite cells/myoblasts are involved in myogenesis, regeneration and muscle fiber repair, it is tempting to hypothesize that such a cytopathic effect may have deleterious consequences on muscle integrity (Figure 3). In particular, a chronic infection could lead, through the cytopathic effect on satellite cells/myoblasts, to a depletion of these progenitor cells during successive cycles of degeneration and regeneration of the muscle fibers.

The persistence of arboviruses in the muscle compartment is a question of major concern [107,108,109]. Indeed, clinical muscular symptoms may remain after the acute phase, e.g., in DENV infection where myalgia persisted in 60% of patients [30], as in SINV six months after infection [31]. Interestingly, concerning the question of persistence, as observed during experimental infection of Cynomolgus macaque with CHIKV, viral RNA could be detected in muscle up to 45 days post-infection [83]. Similarly, during experimental infection of Rhesus macaque with ZIKV, the presence of viral RNA was detected in muscle at 35 days post-infection [90]. Of note, in one patient infected by CHIKV in La Réunion in 2006, viral antigens were detected by immunohistochemistry in the quadriceps, in satellite cells, as late as over 90 days after the acute phase of infection [37].

## 7. Conclusions and Perspectives

Altogether, this review provides insights on the link between arbovirus infection and muscle alterations. If musculo-skeletal signs can frequently be clinically observed during arbovirus infection in humans, this remains often limited within the description of the general symptomatology of Dengue-like state, and has not been specifically investigated. It remains complex to discriminate the specific mechanisms underlying such failure. Animal and cellular models, especially those based on human primary cells, can be of support to investigate at this scope.

Although the studies which have been carried on skeletal muscle infection by arboviruses suggest an etiological link with the observed myopathic syndromes, some aspects of muscle tropism still need to be elucidated; in particular, the exact nature of the target cells must be better characterized for their full susceptibility to arboviruses, e.g., by confirming the presence of specific receptors and/or entry factors in muscle cells, as well as the mechanisms of entry into the cells [105]. Receptors have been suggested for CHIKV, such as prohibition (in microglia cells) [110] or, more recently Mxra [111], as well as for ZIKV, among them AXL [112], which could act on post-entry mechanisms.

Moreover, processes known to be induced by arbovirus infection, such as apoptosis, autophagy, and paraptosis [106,113], need to be better investigated in muscle cells in order to compare them with those observed in other models.

Another peculiar feature of muscle cell susceptibility to arboviral infection is the differential permissiveness of muscle cells according to their state of differentiation, e.g., myoblasts being sensitive to CHIKV infection and not myotubes, whereas myoblasts as well as myotubes seem to be susceptible to Sindbis virus infection. This remains an intriguing point concerning both the differential susceptibility of muscle cells to different arboviruses, as well as the different molecular actors involved in a potential transition from arbovirus susceptibility to resistance to infection during differentiation.

## Figures and Tables

**Table 1:** Ex vivo arbovirus studies in muscle. Arboviruses associated with muscular alteration, inflammation or tropism in human (ex vivo).

Virus (Family, Genus)	Virus (Species)	Clinical Feature	Alteration, Histopathology/Viral Markers (Muscle)	Study	References [*n*°]
***Togaviridae*** ***Alphavirus***	**Chikungunya virus**	myositis	vacuolization, necrosis, infiltrating cells/viral antigens *	La Rèunion case reports (*N* = 2)	[37]
		myositis	sarcoplasmic vacuolization, lymphocytic infiltrate	India case report (*N* = 1)	[55]
	**Sindbis virus**	myalgia (chronic)	internal nuclei, MyHCn+ fibers	Finland case report (*N* = 1)	[31]
***Flaviviridae*** ***Flavivirus***	**Dengue virus**	myalgia	perivascular mononuclear infiltrate, lipid accumulation	Brazil case reports (*N* = 12)	[38]
		myositis	inflammatory cells, fibrotic areas	endemic areas case report (*N* = 1)	[39]
		myositis	perifascicular inflammatory infiltrates, myonecrosis	India case report (*N* = 1)	[42]
		myalgia	myonecrosis, myophagocitosis, inflammation	India case reports (*N* = 2)	[56]
		myopathy	myonecrosis, myophagocytosis	India case reports (*N* = 2)	[57]
		myositis	inflammatory infiltrate	India case reports (*N* = 2)	[58]
		myocarditis † (hemorrhagic fever)	inflammatory markers (MCP-1)/viral antigens #	Colombia case report (*N* = 1)	[59]
		myalgia (hemorrhagic fever) myocarditis †	degeneration of muscle fibers, mononuclear infiltrates/viral antigens-RNA °	Brazil case reports (*N* = 4)	[60]
		myocarditis †	inflammatory infiltrates/viral antigens ^§^	Brazil case reports (*N* = 2)	[61]

* Expression of viral antigens was confirmed in satellite cells. † In case of patients with myocarditis, analysis was performed post-mortem. # MCP-1 antigen was expressed in the endothelium of small myocardial vessels and cardiac interstitial cells, and in myocardial myoblasts; viral antigens were detected in cardiomyocytes, myocardial interstitial and endothelial cells. ° Analysis was performed in heart (i.e., cardiac fibers). ^§^ Expression was confirmed mainly in inflammatory mononuclear cells of myocardium, less in cardiomyocytes.

**Table 3:** In vitro muscle tropism of arboviruses. Arboviruses associated with muscle impairment: cellular models of productive infection in muscle, with description of tropism (in vitro).

Virus (Family, Genus)	Virus (Species)	Cell Study (*N*)	Alteration/Viral Markers, In Vitro Tropism (Muscle Cells)	References [*n*°]
***TogaviridaeAlphavirus***	**Chikungunya virus**	primary myoblasts/satellite cells (human) (2)	cytopathic effect/viral antigens	[37]
		primary myoblasts (human) (3)	cytopathic effect/viral antigens, infectious virions	[94]
		primary myoblasts (human) (4)	cytopathic effect, cytokines production/viral replication	[95]
		myoblasts (murine) myotubes (murine) (2)	viral replication	[80]
		rhabdomyosarcoma (human) (3)	cytopathic effect, cell death/viral replication	[96]
		rhabdomyosarcoma (human) (3)	viral replication	[97]
	**Sindbis virus**	primary myoblasts (human) primary myotubes (human) (2)	cytopathic effect/viral antigens, infectious virions	[31]
***FlaviviridaeFlavivirus***	**Zika virus**	rhabdomyosarcoma (human) (3)	cytopathic effect/viral antigens, viral replication	[98]
		primary myoblasts (human) (3)	cytopathic effect/viral antigens, viral replication	Legros V et al. (accepted)
	**Dengue virus**	primary satellite cells (human) (2)	virus-like particles in cytoplasmic vacuoles/ viral antigens-RNA, viral replication	[99]
		primary myotubes (human)	disregulation of Ca^2+^/homeostasis/viral antigens	[59]
		rhabdomyosarcoma (human) (3)	cytopathic effect/viral antigens, viral replication	[98]
	**West Nile virus**	myoblasts (human embryonic)	viral antigens	[100]
